# A global call to action: strengthening strategies to combat dysglycemia and improve public health outcomes

**DOI:** 10.3389/fpubh.2025.1597128

**Published:** 2025-05-30

**Authors:** Krish Hirani, Brody Sundheim, Mateo Blaschke, Joana R. N. Lemos, Rahul Mittal

**Affiliations:** ^1^Young Leaders Advocacy Group, Diabetes Research Institute Foundation, Hollywood, FL, United States; ^2^Diabetes Research Institute, University of Miami Miller School of Medicine, Miami, FL, United States; ^3^Division of Endocrinology, Diabetes, and Metabolism, Department of Medicine, University of Miami Miller School of Medicine, Miami, FL, United States

**Keywords:** dysglycemia, diabetes, insulin resistance, glycemic control, metabolic disorders, public health policy, economic impact

## Abstract

Dysglycemia, affecting over 800 million people globally, poses a significant challenge to health systems and economies, contributing to healthcare costs exceeding $966 billion annually. This practice paper presents a strategic, multi-level framework addressing dysglycemia through evidence-based interventions. Effective measures include promoting low-glycemic diets, structured physical activity, and continuous glucose monitoring technologies to enhance self-management. Policy initiatives such as sugar taxation and healthy food subsidies incentivize healthier choices, while healthcare provider training enhances clinical capacity. These integrated efforts have demonstrated reduced hospitalizations, improved cognitive function and economic benefits through lower medication costs and enhanced workforce productivity. Addressing healthcare inequities and tailoring interventions to cultural contexts are critical to long-term success.

## Introduction

The rising prevalence of dysglycemia, a condition marked by abnormal blood sugar levels, highlights the urgent need for a comprehensive global response. As an intermediate phase in the progression of diabetes, dysglycemia serves as a critical warning sign, often preceding the onset of the disease. Addressing dysglycemia effectively could help delay or prevent the transition to overt diabetes and mitigate the risk of associated health disorders, including cardiovascular, nephrological disease and neuropathy. A multifaceted approach that combines early detection, lifestyle interventions, and targeted therapies is essential to curb the escalating impact of diabetes worldwide. This alarming trend reflects systemic shortcomings in prevention, early detection, and effective lifestyle interventions. Without decisive, evidence-based strategies, the global health and economic burden of dysglycemia will continue to grow unabated.

Several interconnected factors are driving this rapid increase in dysglycemia. Lifestyle shifts, including a reliance on ultra-processed, high-sugar diets and sedentary behaviors, have significantly contributed to rising obesity rates and insulin resistance. Urbanization has further exacerbated these trends, as reduced physical activity becomes a hallmark of modern city living (1). Additionally, aging populations, with declining metabolic function, are increasingly predisposed to impaired glycemia. Socioeconomic inequities also play a critical role, with limited access to healthcare, nutritious food, and early screening disproportionately affecting low- and middle-income countries, where diabetes prevalence is climbing fastest. Genetic and epigenetic factors compound these challenges, amplifying susceptibility in vulnerable populations when combined with environmental triggers (2).

Obesity is a significant risk factor for dysglycemia, encompassing a range of glucose metabolism disorders (3–5). Its prevalence is intricately linked to socioeconomic status (SES) and nutritional behaviors (6–8). In high-income countries, individuals with lower SES often exhibit higher rates of obesity and dysglycemia, attributed to factors such as limited access to healthy foods, reduced opportunities for physical activity, and increased exposure to stressors that can lead to unhealthy eating habits (9). Conversely, in low- and middle-income countries, higher SES is sometimes associated with increased obesity rates, possibly due to greater consumption of energy-dense foods and sedentary lifestyles (10, 11). A study analyzing data from the U.S. National Health and Nutrition Examination Survey (NHANES) found that lower SES was associated with higher diabetes risk, mediated by factors like reduced physical activity and limited access to healthcare services (12). Another study suggested that individuals from lower SES backgrounds often consume diets higher in fats and simple carbohydrates while lacking in fruits, vegetables, and whole grains, contributing to increased obesity and dysglycemia risk (13). Addressing these disparities requires multifaceted interventions that consider the socioeconomic and environmental contexts influencing dietary behaviors and health outcomes.

The implications of obesity and dysglycemia are profound. Healthcare systems are struggling under the weight of diabetes-related complications, including cardiovascular diseases, renal failure, and neuropathy, leading to strained resources and overwhelmed infrastructures (14). Economically, the costs associated with diabetes management and its complications are projected to surpass $1 trillion USD globally by 2030 (15). Workforce productivity is also at risk, as diabetes-related disability, absenteeism, and premature mortality increase dependency ratios and hinder economic growth (16).

Urgent action is required to prevent the detrimental effects of dysglycemia. Effective interventions include community education programs to promote health literacy, dietary awareness, and sustainable lifestyle changes (Table 1). Policy reforms, such as sugar taxation, food labeling regulations, and subsidies for nutritious foods, are essential to creating an environment conducive to healthier choices (Table 1). Expanding access to screening and early detection tools is equally critical to mitigating disparities and ensuring timely interventions. International cooperation, informed by evidence-based frameworks, is indispensable for addressing the global diabetes crisis and building resilient healthcare systems capable of meeting this challenge (17).

**Table 1:** Overview of challenges associated with dysglycemia, proposed solutions, and their expected impact.

Challenge	Proposed solution	Impact
Rising prevalence of dysglycemia	Lifestyle interventions and early screening programs	Reduced diabetes incidence and improved early detection
Economic burden of healthcare costs	Sugar taxes, subsidies, and workplace wellness initiatives	Lower costs, increased productivity, and sustainability
Inequitable healthcare access	Telemedicine platforms, affordable medications, and community programs	Expanded access to preventive care and monitoring
Slow adoption of new technologies	Investment in glucose monitoring, AI tools, and precision medicine	Personalized treatment strategies and cost-efficiency
Gaps in public awareness and education	Mass media campaigns and cultural adaptations for diverse populations	Increased awareness and behavioral changes

## The damaging effects of high blood sugar on physical and mental health

Chronic hyperglycemia, characterized by prolonged elevated blood sugar levels, has devastating effects on multiple organ systems, significantly increasing the risk of life-threatening diseases. The cardiovascular system is particularly vulnerable, with excessive glucose contributing to endothelial dysfunction, atherosclerosis, hypertension, and an increased risk of stroke and heart attacks. High blood sugar leads to oxidative stress and chronic inflammation, damaging arterial walls and impairing circulation, which can result in peripheral artery disease (PAD) and heart failure (18).

The renal system is another major target, as prolonged hyperglycemia causes microcirculatory damage to the kidneys, leading to diabetic nephropathy, a progressive condition that can result in end-stage renal disease (ESRD), necessitating dialysis or transplantation (19). Excess glucose triggers the production of advanced glycation end products (AGEs), which accelerate kidney fibrosis and decline function.

The nervous system is severely affected by hyperglycemia, with both peripheral and autonomic neuropathy developing over time. Diabetic neuropathy manifests as pain, tingling, and loss of sensation in the extremities, increasing the risk of foot ulcers and amputations. Autonomic nervous system dysfunction leads to gastrointestinal, cardiovascular, and bladder control issues (20).

The endocrine and metabolic systems suffer under prolonged hyperglycemia, with insulin resistance contributing to metabolic syndrome, obesity, and an increased susceptibility to type 2 diabetes. This dysregulation of glucose homeostasis further aggravates dyslipidemia, characterized by altered lipid profiles, thereby heightening the risk of metabolic dysfunction-associated steatotic liver disease (MASLD) and impairing pancreatic exocrine function (21).

Ocular complications such as diabetic retinopathy, cataracts, and glaucoma are well-documented effects of high blood sugar. Persistent hyperglycemia damages the retinal microvasculature, leading to vision loss and blindness in severe cases (22).

In the immune system, high glucose levels impair white blood cell function, reducing the body’s ability to fight infections and increasing susceptibility to bacterial and fungal infections. Poor wound healing and recurrent infections are common in individuals with poorly controlled blood sugar (23).

The mental health impact of high blood sugar is profound. Studies show that chronic hyperglycemia is linked to an increased risk of depression, anxiety, and cognitive decline, including Alzheimer’s disease. Elevated blood sugar levels contribute to neuroinflammation and oxidative stress, accelerating brain aging and impairing neurotransmitter function, which negatively affects memory, learning, and emotional regulation (24).

Moreover, the gut-brain axis is influenced by hyperglycemia, with evidence suggesting that poor glycemic control alters gut microbiota composition, leading to systemic inflammation and further exacerbation of mental health disorders (25).

Overall, the scientific consensus is clear: chronic hyperglycemia imposes a significant burden on health and well-being, with evidence-backed interventions needed to prevent and mitigate its widespread consequences.

## Understanding the challenge

### Physiological disruptions

Dysglycemia arises from disruptions in glucose regulation, involving intricate interactions between the pancreas, liver, skeletal muscles, and nervous system. The pancreas plays a central role by producing insulin and glucagon, hormones crucial for maintaining blood glucose levels. Dysglycemia occurs when insulin secretion or its action becomes impaired, leading to hyperglycemia or hypoglycemia (26). The liver, responsible for glucose storage and release, can exacerbate this imbalance by overproducing glucose during insulin resistance. Skeletal muscles, a primary site for glucose uptake, may exhibit impaired glucose utilization due to defects in insulin signaling pathways, particularly those affecting the translocation and function of glucose transporter type 4 (GLUT4). Dysregulation of the insulin receptor substrate (IRS) and phosphoinositide 3-kinase (PI3K)/Akt signaling cascade can significantly impair GLUT4 trafficking to the plasma membrane, thereby disrupting glucose homeostasis. Furthermore, the nervous system, especially the autonomic and peripheral nerves, plays a pivotal role in regulating glucose metabolism through complex neuroendocrine mechanisms, including modulation of insulin secretion and sensitivity via the hypothalamic–pituitary–adrenal (HPA) axis and sympathetic nervous system. Dysglycemia induces oxidative stress, chronic inflammation, and mitochondrial dysfunction, all of which further damage these systems and perpetuate a cycle of metabolic dysregulation (27).

### Systemic impact

The systemic impact of dysglycemia is profound, affecting nearly every organ system. Chronic hyperglycemia accelerates aging by increasing the production of advanced glycation end products (AGEs), which impair cellular and tissue function. Cognitive decline, including dementia and mild cognitive impairment, is linked to chronic dysglycemia, as the brain relies heavily on regulated glucose supply. Additionally, dysglycemia significantly increases the risks of chronic diseases such as cardiovascular disorders, kidney failure, and certain cancers. The cumulative burden of these complications reduces quality of life and places immense strain on healthcare systems globally (28–30).

### Roadblocks to optimal glucose control

Barriers to achieving glycemic stability are deeply rooted in social determinants of health. Limited access to nutritious food and a growing reliance on inexpensive, ultra-processed, and sugary products contribute to poor dietary habits. Sedentary lifestyles, driven by urbanization and lack of physical activity opportunities, further exacerbate the problem. In underserved regions, limited access to healthcare services, preventive screenings, and educational resources hinders early detection and management of dysglycemia, disproportionately impacting vulnerable populations (31).

### Need for comprehensive solutions

Addressing dysglycemia requires a multifaceted approach that combines individual, community, and systemic strategies. Individual efforts, such as promoting dietary changes and regular physical activity, need to be supported by community-driven programs that create supportive environments for healthy behaviors. On a systemic level, policy reforms that improve healthcare access, implement sugar taxes, and regulate food marketing are essential to addressing structural barriers. By integrating these approaches, it is possible to achieve glycemic stability, mitigate health risks, and alleviate the economic and social burden of dysglycemia worldwide (32).

### Empowering individuals

#### Dietary interventions

Promoting dietary changes is a cornerstone of managing dysglycemia, with a focus on macro and micronutrient composition and glycemic response modulation (33). The incorporation of low-glycemic index (GI) foods and meals, characterized by gradual carbohydrate absorption and lower postprandial glucose excursions, has been shown to improve insulin sensitivity and reduce HbA1C levels, including patients with established T1D (34). Foods such as legumes, whole grains, and non-starchy vegetables delay gastric emptying and promote a more stable release of glucose, primarily through their fiber content and slower digestion rates (35). Moreover, the inclusion of polyunsaturated fatty acids (PUFAs), such as omega-3 (eicosapentaenoic acid and docosahexaenoic acid) and omega-6 fatty acids – observing the right ratio, has demonstrated efficacy in enhancing insulin receptor signaling by incorporating into cell membrane phospholipids, thereby improving membrane fluidity and downstream insulin signaling cascades, particularly the PI3K/Akt pathway. Monounsaturated fatty acids (MUFAs), predominantly found in olive oil, avocados, and nuts, exert beneficial effects on dysglycemia by reducing oxidative stress and inflammation through the modulation of nuclear factor-kappa B (NF-κB) and activation of peroxisome proliferator-activated receptors (PPARs) (36). Additionally, polyphenol-rich diets, comprising flavonoids and anthocyanins, further support glucose homeostasis by inhibiting alpha-glucosidase, enhancing endothelial function, and modulating gut microbiota composition (37). The synergistic effects of these dietary components underscore the potential of personalized nutrition tailored to cultural and regional preferences to ensure widespread adoption. Strategies in mitigating dysglycemia and preventing its progression to diabetes. For instance, local food staples have been integrated into diet plans to make healthier choices accessible and affordable. Educational campaigns focused on portion control, nutritional literacy, and the long-term benefits of balanced meals have been pivotal in encouraging sustainable eating habits. By addressing socioeconomic barriers to accessing fresh and nutritious foods, these interventions not only support individuals but also create ripple effects across communities (38, 39).

Emerging evidence highlights the significant influence of nutrition and food literacy on glycemic control, particularly concerning HbA1c levels in individuals with diabetes. A randomized controlled trial in Thailand demonstrated that a Collaborative Learning-Based Food Literacy Enhancement Program (CLFLEP) led to substantial improvements in healthy eating behaviors and reductions in HbA1c among older adults with uncontrolled type 2 diabetes (40). The findings of this study highlight the effectiveness of structured food literacy interventions in enhancing metabolic outcomes. Similarly, a study found that patients with higher food literacy scores experienced more favorable changes in HbA1c levels compared to those with lower scores, emphasizing the role of food literacy in glycemic management (41). Additionally, research has shown that individuals with inadequate health literacy tend to have higher HbA1c levels and poorer lipid profiles, suggesting that health literacy is a critical determinant of diabetes control (42). Collectively, these findings suggest the necessity of incorporating nutrition and food literacy education into diabetes care strategies to improve glycemic control and overall health outcomes.

#### Physical activity programs

Structured exercise programs have demonstrated significant benefits in reducing insulin resistance and improving glucose metabolism. Community-led initiatives, such as walking groups and fitness clubs, provide accessible and low-cost opportunities for physical activity. Workplace wellness programs have been implemented to encourage employees to incorporate movement into their daily routines, while school-based physical education programs instill healthy habits early in life. The scalability of these programs makes them ideal for large-scale adoption, ensuring that physical activity becomes a regular part of individuals’ lives (43–45).

#### Technology integration

The integration of continuous glucose monitoring (CGM) systems has revolutionized personal glycemic management. These devices provide real-time data on glucose levels, allowing individuals to make informed decisions about their diet, exercise, and medication. By fostering a sense of autonomy and accountability, CGM systems empower individuals to actively participate in managing their health. Additionally, mobile health apps and wearable technologies further enhance these systems, offering personalized feedback and promoting long-term adherence to glycemic control strategies (46, 47).

### Building clinical capacity

#### Healthcare provider training

Equipping healthcare professionals with knowledge and tools to manage dysglycemia is essential for improving patient outcomes. Comprehensive training programs have focused on evidence-based, patient-centered care, enabling providers to offer personalized treatment plans that address each patient’s unique needs. These programs include workshops, certifications, and continuous medical education modules that emphasize the latest advancements in glycemic management. By strengthening clinical expertise, these initiatives have enhanced the overall quality of care and patient satisfaction (48–50).

#### Community engagement

Building strong partnerships between clinicians and community leaders has been instrumental in raising awareness about dysglycemia and encouraging community participation in health initiatives. Local leaders, trusted by their communities, serve as advocates for preventive measures such as early screenings, healthy eating, and regular exercise. Collaborative events, such as health fairs and awareness campaigns, provide platforms for disseminating information and promoting active engagement (51, 52).

#### Localized support

Culturally adapted counseling services and group education sessions have been vital in addressing patient concerns and fostering behavioral change. For example, diabetes educators work closely with individuals and families to provide tailored advice that aligns with cultural norms and dietary practices. Group sessions create supportive environments where individuals can share their experiences, learn from peers, and stay motivated to adhere to treatment plans. These localized efforts bridge gaps in healthcare delivery, ensuring that interventions are relevant, relatable, and effective (53, 54).

### Systemic policy reforms to enable change

#### Sugar taxes

The implementation of sugar taxes has proven to be a highly effective strategy for reducing the consumption of sugary beverages and processed foods. By increasing the cost of these products, governments disincentivize their purchase, encouraging consumers to opt for healthier alternatives. One of the most well-documented examples is Mexico’s sugar tax, introduced in 2014, which led to an average 7.6% decline in sugary drink purchases in the first year and a 10.2% reduction among low-income households, groups most vulnerable to diet-related non-communicable diseases (55–58). In the United Kingdom, the Soft Drinks Industry Levy prompted manufacturers to proactively reduce sugar content in beverages, resulting in a 29% drop in total sugar purchased from soft drinks between 2015 and 2018 (59).

Other countries have also reported measurable improvements in public health outcomes (60–65). The implementation of South Africa’s Health Promotion Levy (HPL) in April 2018 has been associated with significant reductions in sugar consumption from sugar-sweetened beverages (SSBs) (66). A study analyzing household purchase data from 2014 to 2019 found that the mean daily sugar intake from taxable beverages decreased from 16.25 grams per capita before the HPL announcement to 10.63 grams per capita after its implementation (66). Additionally, the volume of taxable beverage purchases declined from 518.99 mL to 443.39 mL per capita per day over the same period. These reductions were more pronounced among lower socioeconomic groups, indicating the HPL’s potential to improve health equity. In Chile, the implementation of the Law of Food Labeling and Advertising in 2016, which introduced mandatory front-of-package warning labels for products high in sugars, sodium, saturated fats, or calories, resulted in a significant reduction in the consumption of unhealthy beverages (67). A study evaluating purchases from 2015 to 2017 found a 23.7 percent decline in the volume of labeled “high-in” beverage purchases, corresponding to a reduction of 22.8 milliliters per capita per day. Additionally, there was a 27.5% decrease in calories purchased from these beverages (67). These reductions were observed across all education levels, indicating the broad population-level impact of the policy. The study suggests that comprehensive labeling laws can effectively shift consumer behavior and contribute to improved dietary patterns and public health outcomes. The Philippines’ sugar-sweetened beverage tax, also enacted in 2018, produced a marked decline in sugary drink sales while simultaneously boosting water and unsweetened beverage consumption (64).

In the United States, local interventions have similarly demonstrated success. In Berkeley, California, the implementation of a local sugar tax in 2015 resulted in a 9.6% reduction in sugary drink consumption in the first year, accompanied by a 15.6% increase in water intake (68). These diverse case studies collectively illustrate that fiscal measures such as sugar taxation can effectively drive both consumer behavior change and industry reformulation, contributing to reduced caloric intake from added sugars and lowering the overall burden of dysglycemia and other non-communicable diseases. As more regions adopt such policies, their potential to promote healthier lifestyles and reduce the burden of non-communicable diseases continues to grow (69, 70).

#### Healthy food subsidies

To complement sugar taxes, subsidies for nutritious foods have been introduced to promote equitable access to healthy diets. Programs such as the U.S. Supplemental Nutrition Assistance Program (SNAP) provide financial incentives for purchasing fruits, vegetables, and whole grains, making these options more affordable for low-income families. In Brazil, government-supported initiatives have linked local farmers to urban food distribution systems, ensuring the availability of fresh produce in underserved areas. By reducing the cost barrier to healthy eating, these subsidies address socioeconomic disparities in diet quality and support long-term health improvements (70–74).

Other countries have also implemented similar initiatives with success. In the United Kingdom, the Healthy Start scheme provides low-income families with vouchers to purchase fresh fruits, vegetables, milk, and formula, improving maternal and child nutrition. Finland’s fruit and vegetable subsidy program has encouraged healthier eating habits, leading to significant increases in fruit and vegetable consumption, particularly among children and adolescents (75–78).

In India, the Public Distribution System (PDS) subsidizes essential staples like rice, wheat, and pulses for low-income populations while piloting programs to include nutrient-rich foods such as fortified grains and edible oils. Rwanda’s Girinka program provides smallholder farmers with livestock subsidies, indirectly boosting dietary diversity by increasing access to animal-source foods like milk and eggs (79–81).

In Australia, remote indigenous communities benefit from subsidies on healthy foods through programs like the Outback Stores initiative, which ensures affordable prices for fresh produce in isolated areas. These global examples highlight how strategic subsidies can improve diet quality, reduce health disparities, and promote sustainable, healthier eating behaviors across diverse populations (82, 83).

#### Innovative strategies

Besides sugar taxes and healthy food subsidies, innovative strategies are emerging to promote equitable access to healthy diets and transform food environments. One novel concept is “produce prescription programs,” which integrate healthcare and nutrition by allowing physicians to “prescribe” fresh fruits and vegetables as part of medical treatment plans. These prescriptions are often redeemable for free or discounted produce through partnerships with local grocery stores and farmers’ markets. Early implementations in the United States, such as the Fresh Food Pharmacy initiative, have demonstrated success in improving dietary intake and glycemic control among patients with diabetes (84–87).

Another cutting-edge approach is the development of “smart subsidies” powered by artificial intelligence (AI) and data analytics. These programs use AI to analyze individual purchasing behaviors and provide personalized discounts or incentives for healthier choices directly at the point of sale. For example, a pilot program in Europe used machine learning algorithms to identify high-sugar product purchases and automatically apply discounts to healthier alternatives during checkout, effectively nudging consumers toward better options (88, 89).

A particularly innovative concept is the “health footprint tax,” which extends beyond sugar to include the environmental and health impacts of food production and consumption. This approach assigns taxes or incentives based on a food’s combined impact on health and sustainability. For instance, ultra-processed foods with high environmental costs and negative health outcomes would incur higher taxes, while locally sourced, minimally processed, nutrient-rich foods would receive subsidies (90, 91).

Finally, the use of behavioral economics in school cafeterias, known as “choice architecture,” is gaining traction. By strategically placing healthier options at eye level or offering smaller portions of unhealthy foods as defaults, researchers have found significant improvements in dietary choices among children. This approach capitalizes on subconscious decision-making to instill healthier eating habits early in life (92, 93).

These novel strategies push the boundaries of traditional public health measures, offering transformative potential to improve diets, reduce chronic disease burdens, and ensure equity in food systems. They also emphasize the importance of integrating technology, behavioral science, and cross-sector collaboration into modern nutrition policy frameworks.

### Regulatory policies to create healthier environments

#### Marketing restrictions

Restricting the marketing of unhealthy foods, particularly to children, is a crucial policy intervention. Children are highly susceptible to advertising, and exposure to marketing for high-sugar, high-fat, and highly processed products influences their preferences and eating habits. Countries such as Norway and Chile have implemented strict regulations banning junk food advertisements during children’s programming and requiring clear warning labels on unhealthy products. These measures have reduced the appeal of such products and encouraged families to make healthier food choices. Limiting exposure to these advertisements protects vulnerable populations and reduces the normalization of unhealthy diets (94, 95).

#### Public-private partnerships

Collaborations between governments and the private sector have played a pivotal role in reformulating products and improving nutritional labeling. For instance, initiatives like the European Union’s salt reduction framework have engaged food manufacturers in reducing salt levels in processed foods while maintaining taste and consumer acceptance. Similarly, partnerships in the U.S. have led to the introduction of front-of-pack labeling systems that highlight nutritional information, enabling consumers to make more informed choices. These collaborations align industry interests with public health goals, fostering a collective effort to improve population health (96, 97).

#### Infrastructure improvements

Physical environments significantly influence lifestyle behaviors, and improving access to recreational facilities and walkable urban designs can promote healthier living. Investments in public parks, bike lanes, and pedestrian-friendly infrastructure encourage physical activity and reduce sedentary behavior. For example, the Netherlands’ extensive cycling infrastructure has been linked to lower obesity rates and better cardiovascular health. In the U.S., initiatives such as the “Complete Streets” program prioritize designing streets that accommodate pedestrians, cyclists, and public transit users. These efforts create environments that support active lifestyles, ultimately contributing to better glycemic control and overall health (98).

### Impact and lessons learned

Economic considerations play a pivotal role in the global effort to combat dysglycemia. Preventive programs and innovative interventions not only improve glycemic control but also yield substantial cost savings for individuals and healthcare systems. The key interventions, their associated cost savings, and references to studies that demonstrate their effectiveness has been summarized in Table 2. These insights highlight the economic feasibility of scaling these measures to reduce the financial burden of dysglycemia-related complications while enhancing overall health outcomes.

**Table 2:** Estimated cost savings from specific interventions aimed at managing dysglycemia.

Intervention	Cost savings
Preventive screening programs	Early detection saves $3,000–$5,000 per patient annually.
Medication subsidies	Reduces polypharmacy dependence, lowering long-term medication costs.
Workplace wellness programs	Saves $90 billion/year by improving productivity and reducing absenteeism.
Sugar taxes	Reduces sugar consumption and generates revenues for health programs.
Community-based programs	Low-cost interventions improve health behaviors and screening rates.
Telemedicine and remote monitoring	Expands access to care, reducing hospitalizations and emergency visits.

#### Tangible outcomes

One of the most significant impacts has been the reduction in hospital admissions related to diabetes complications, such as cardiovascular events, renal failure, and neuropathy. By promoting preventive measures, such as lifestyle changes and early screenings, healthcare systems have experienced a tangible decrease in the burden of acute and chronic complications. Participants in community-based glycemic control programs have shown improved glycemic stability, evidenced by lower HbA1c levels and reduced incidence of hyperglycemic and hypoglycemic episodes. Additionally, enhanced glycemic control has translated to better physical and cognitive health, enabling individuals to remain active contributors to the workforce. Employers have reported improved productivity and reduced absenteeism, underscoring the broader societal benefits of addressing dysglycemia (99, 100).

#### Economic benefits

Cost-effectiveness analyses have highlighted the economic advantages of glycemic control initiatives. Lower medication costs, resulting from better glycemic stability and fewer complications, have significantly reduced the financial burden on both individuals and healthcare systems. For instance, implementing continuous glucose monitoring (CGM) technologies and subsidizing healthy foods have demonstrated substantial returns on investment by preventing costly interventions for advanced diabetes-related conditions. Fewer hospitalizations and disability claims have further alleviated economic strain, while productivity gains from healthier workforces have contributed to sustained economic growth. These findings emphasize the long-term economic viability of investing in preventive and management-focused dysglycemia programs (101).

#### Healthcare equity

Addressing healthcare disparities has been a critical focus of these initiatives, with efforts aimed at reaching underserved populations who face the highest risks of dysglycemia and its complications. Expanding access to affordable technologies, such as CGMs and early diagnostic tools, has been instrumental in reducing inequities. Subsidized programs for low-income families have improved access to nutritious foods and healthcare services, enabling vulnerable groups to participate in glycemic control interventions. However, persistent gaps in access remain a challenge, highlighting the need for continued investment in equitable healthcare delivery models (102).

### Challenges and adaptations

Despite the successes, several hurdles have been encountered in implementing these interventions. Cultural resistance to dietary changes, for example, posed challenges in some communities where traditional diets are high in refined carbohydrates and sugars. To overcome this, culturally sensitive education programs and localized food substitutions were introduced to align interventions with regional preferences. Logistic barriers, such as limited healthcare infrastructure in rural areas, also hindered the reach of programs. Mobile health clinics and telemedicine solutions were deployed to bridge these gaps, ensuring that underserved populations had access to essential services. Additionally, the adoption of new technologies, such as CGMs, required extensive training for both patients and healthcare providers, which was addressed through dedicated workshops and educational materials (103).

### Policy recommendations: a roadmap for action

To address the growing burden of dysglycemia globally, policymakers must adopt a multi-sectoral, evidence-based approach that integrates fiscal measures, healthcare system strengthening, regulatory frameworks, and community engagement. A comprehensive policy roadmap must prioritize both prevention and management strategies while ensuring equitable access to healthcare and healthy lifestyle choices across populations.

A crucial component of this policy roadmap is the implementation of fiscal policies such as sugar taxation and food subsidies. Countries such as Mexico have demonstrated the effectiveness of taxation policies on sugar-sweetened beverages, leading to a 6% decline in consumption within the first year of implementation and a 10% decline among lower-income households (104). Such measures generate revenue that can be reinvested into public health initiatives, including nutrition education and preventive healthcare services. Simultaneously, subsidies for healthier food alternatives, such as whole grains and fresh produce, can improve affordability and access, particularly in low- and middle-income countries where nutritious food is often cost-prohibitive (72).

Healthcare infrastructure development is also critical for enhancing glycemic control interventions. Expanding the availability of continuous glucose monitoring (CGM) systems and telemedicine services can bridge accessibility gaps, particularly in rural and underserved areas. Studies have shown that integrating CGM into primary care settings leads to improved glycemic outcomes and reduces hospitalization rates by up to 40% (105). Moreover, workforce capacity must be strengthened through comprehensive training programs that equip healthcare providers with the latest evidence-based strategies for dysglycemia management, including personalized treatment approaches and behavior change counseling.

Regulatory frameworks play a significant role in shaping healthier environments. Front-of-package food labeling policies, as adopted in countries such as Chile, have successfully influenced consumer behavior by clearly indicating products high in sugar, sodium, and saturated fats, resulting in a decline in unhealthy food purchases (67). Similarly, restricting the marketing of ultra-processed foods to children has been effective in reducing their exposure to unhealthy dietary influences, thus helping to prevent early onset of dysglycemia and related metabolic disorders (106).

Community engagement remains a cornerstone of effective policy implementation. Public health campaigns that promote dietary literacy and physical activity have been instrumental in countries like Finland, where targeted nutrition programs have significantly reduced the prevalence of metabolic disorders over the past two decades (107). Engaging local stakeholders, such as schools, workplaces, and religious institutions, can foster culturally tailored interventions that resonate with diverse populations, ensuring sustainability and long-term impact.

Evidence-driven policymaking is essential for tracking progress and optimizing interventions. The use of artificial intelligence (AI) and big data analytics enables policymakers to monitor trends in dysglycemia prevalence, evaluate the effectiveness of interventions, and adjust policies accordingly. The United Kingdom’s National Health Service (NHS) has successfully leveraged data analytics to enhance population health strategies, improving early detection rates and personalized treatment plans (108).

### Case studies: successful national-level interventions

Several countries have successfully implemented national-level interventions that provide valuable insights and best practices for tackling dysglycemia on a global scale.

Finland’s comprehensive nutrition policy stands out as a model for integrating public health and food systems. Through a combination of fiscal measures, education campaigns, and regulatory initiatives, Finland has significantly reduced the prevalence of cardiovascular diseases linked to poor glycemic control. Key strategies include the introduction of school meal programs that provide nutritionally balanced meals and the implementation of salt and sugar reduction programs across the food industry (109). These interventions have contributed to improved population-wide dietary habits and better health outcomes.

In Brazil, the National Food and Nutrition Policy focuses on improving access to fresh and minimally processed foods, particularly for low-income communities. The policy supports smallholder farmers through urban distribution programs and encourages dietary diversity through national dietary guidelines that emphasize traditional and minimally processed foods (110). These efforts have contributed to a gradual improvement in diet quality and glycemic control among the Brazilian population.

## Future directions: research and collaboration opportunities

Future research efforts should focus on personalized medicine approaches to dysglycemia prevention and management. The integration of genetic, microbiome, and lifestyle data can facilitate the development of individualized treatment plans that optimize glycemic control and reduce the risk of complications. Studies exploring the role of gut microbiota in glucose metabolism, for instance, offer promising insights into novel therapeutic targets (111).

Technology-driven interventions present significant opportunities for innovation in dysglycemia management. The advancement of AI-powered digital health tools can enhance real-time glucose monitoring, provide personalized dietary recommendations, and predict glycemic fluctuations based on lifestyle patterns (112). Expanding access to these technologies in low-resource settings remains a critical area for further exploration.

Cross-sector collaboration is essential to scaling successful interventions globally. Partnerships between governments, non-governmental organizations, academic institutions, and the private sector can facilitate knowledge-sharing, and the implementation of best practices tailored to diverse contexts. International frameworks such as the WHO Global Diabetes Compact provide opportunities for coordinated action and alignment of national policies with global targets (113).

Equity-focused research is crucial to addressing healthcare disparities in dysglycemia prevention and treatment. Studies should explore the impact of social determinants of health, including access to nutritious food, healthcare services, and education, to inform policies that promote equitable access to care across populations. Tailoring interventions to culturally specific needs and socioeconomic contexts will be critical in achieving sustainable health improvements (114).

Sustainability considerations must also be integrated into future strategies. The intersection of environmental sustainability and food systems presents an opportunity to promote dietary patterns that are both health-promoting and environmentally sustainable, such as plant-based diets with low environmental footprints (115).

## Conclusions and call to action

Addressing the global burden of dysglycemia requires the coordinated efforts of diverse stakeholders, as summarized in Table 3.

**Table 3:** Stakeholder roles and actionable strategies for improving glycemic control.

Stakeholder group	Actionable strategies	Expected impact
Healthcare providers	1. Integrate routine glycemic screening and lifestyle counseling into clinical practice.	Early detection and prevention of complications.
	2. Expand access to telemedicine platforms for remote consultations and glucose monitoring.	Improved accessibility and patient adherence, particularly in underserved areas.
	3. Offer personalized treatment plans, including pharmacological and lifestyle-based approaches.	Optimized glycemic control and reduction in disease progression.
Policymakers	1. Increase funding for public health campaigns and school-based programs promoting healthy diets and physical activity.	Enhanced awareness, early prevention, and reduced childhood obesity rates.
	2. Implement sugar taxes, food labeling reforms, and subsidies for healthy foods to encourage dietary changes.	Reduced sugar consumption, healthier food choices, and decreased diabetes incidence.
	3. Expand access to affordable medications and monitoring technologies through subsidies and insurance reforms.	Broader healthcare access, cost savings, and improved health equity.
Researchers and innovators	1. Develop predictive biomarkers and genetic screening tools to identify high-risk populations.	Early diagnosis and prevention through precision medicine approaches.
	2. Focus on pharmacological advancements, including GLP-1 receptor agonists, SGLT2 inhibitors, and anti-inflammatory therapies.	Improved treatment outcomes and reduced complications.
	3. Create AI-driven analytics platforms and digital monitoring tools for real-time glycemic tracking and intervention adjustments.	Enhanced disease management and resource optimization.
Public and private sectors	1. Establish multi-sector partnerships to fund and implement public health programs, including community fitness centers and mobile screening units.	Scalable and sustainable interventions targeting diverse populations.
	2. Leverage big data and AI technologies to track trends, assess policy effectiveness, and refine implementation strategies.	Data-driven policy enhancements and efficient allocation of resources.
	3. Promote workplace wellness programs and incentives for physical activity to encourage healthier lifestyles.	Reduced absenteeism, higher productivity, and improved workforce health outcomes.

The evidence presented demonstrates that a multi-pronged approach integrating community-driven interventions with systemic policy reforms can significantly improve outcomes. Empowering individuals through dietary changes, physical activity, and access to innovative technologies, combined with fiscal measures and regulatory policies, has shown measurable success in achieving glycemic stability, reducing complications, and alleviating economic burdens. These efforts highlight the importance of collaboration across all levels of society to combat this growing epidemic.

The undeniable value of combining grassroots, community-driven approaches with structural, systemic reforms emerges as the key lesson from these initiatives. Systemic changes, such as sugar taxes, healthy food subsidies, and marketing regulations, create environments that support and sustain healthy behaviors. Together, these strategies address immediate challenges while laying the foundation for long-term health improvements and economic resilience.

To replicate these successes globally, strategies must be adapted to the cultural, economic, and social contexts of diverse populations. Dietary interventions should align with local food habits, while fiscal measures such as sugar taxes must be tailored to regional economic structures. Efforts to expand access to health technologies must prioritize low- and middle-income countries, where healthcare disparities are most pronounced. By learning from successful models and customizing interventions to local needs, these strategies can be scaled effectively across the globe.

A coordinated effort involving policymakers, healthcare providers, researchers, and community leaders is essential to address the multifaceted challenge of dysglycemia. Policymakers must prioritize equitable access to healthcare and implement evidence-based reforms, while healthcare providers play a crucial role in delivering patient-centered care and education. Researchers, in turn, must continue to innovate and evaluate interventions to ensure their effectiveness and scalability. Collaboration across sectors and regions will not only strengthen the fight against dysglycemia but also foster partnerships that promote health equity and sustainable development.

The fight against dysglycemia represents a unique opportunity to redefine global health priorities. By embracing innovative and inclusive strategies, we can achieve better metabolic health for millions, reduce the economic burden of chronic diseases, and support healthy aging on a global scale. These efforts will enhance individual and community well-being and contribute to the development of resilient health systems and sustainable economies. Through collective action, this aspirational goal is within reach.
